# Factors associated with frozen shoulder in adults: a retrospective study

**DOI:** 10.1186/s12891-024-07614-8

**Published:** 2024-06-26

**Authors:** Xiarepa Abudula, Palida Maimaiti, Ailiyaer Yasheng, Jiaojiao Shu, Asiguli Tuerxun, Halimire Abudujilili, Ruiqi Yang

**Affiliations:** 1 Nursing School of Xinjiang Medical University, Urumqi, 830054 China; 2 Hospital of Xinjiang Traditional Uyghur Medicine, Urumqi, China

**Keywords:** Frozen shoulder, Diabetes, Gender, Risk Factors

## Abstract

**Objective:**

This study aims to explore the risk factors associated with frozen shoulder patients and further analyze the relationship between gender and diabetes with frozen shoulder.

**Method:**

We have reviewed the data of 1205 frozen shoulder patients in China's Xinjiang region from 2018 to 2023. The collected information included patients' gender, occupation, place of origin, marital status, age, the season of disease onset, duration of illness, etiology, surgical history, hypertension, diabetes, respiratory diseases, knee joint disease, hyperlipidemia, cardiovascular diseases, cervical spondylosis, lumbar disc herniation, rheumatoid arthritis, hyperuricemia, sleep quality, smoking and alcohol consumption, and constipation. We have used multifactor logistic regression analysis to identify the risk factors for a frozen shoulder.

**Results:**

Single-factor logistic regression analysis showed that the number of females, patients with diabetes, knee joint disease, constipation, and patients with poor sleep quality in the observation group are higher than in the control group (*P* < 0.05). There were no statistically significant differences between the two groups in terms of occupation, place of origin, marital status, age, season of disease onset, duration of illness, etiology, surgical history, hypertension, respiratory diseases, hyperlipidemia, cardiovascular diseases, cervical spondylosis, lumbar disc herniation, rheumatoid arthritis, hyperuricemia, smoking, and alcohol consumption history (*P* > 0.05).

Multivariate analysis showed that the final model included four variables: gender, diabetes history, sleep, and constipation. Among them, the OR values of gender and diabetes history were more significant than 1, indicating that they were independent risk factors for frozen shoulder, while the OR values of sleep and constipation were less than 1, suggesting that they were negatively associated with the occurrence of frozen shoulder.

**Conclusion:**

The results of this study suggest that gender and diabetes are independent risk factors for frozen shoulder. Additionally, poor sleep quality and constipation also can be correlated with the occurrence of a frozen shoulder.

## Introduction

Frozen shoulder (FS) is a common shoulder disorder characterized by shoulder pain, stiffness, and limited range of motion [1]. Frozen shoulder is a chronic and specific inflammatory condition characterized by pain and limited mobility, which may lead to atrophy of the shoulder muscles due to disuse [2, 3]. It is normally classified into inflammatory and structural origin [4]. Throughout the condition period, there is persistent pain and a gradual decrease in the passive and active range of motion in the shoulder [5]. In the general population, the prevalence rate is as high as 8.2% in males and 10.1% in females, with a peak incidence at 55 years of age [6]. FS can severely impact the patient’s daily life and workability, and it has more adverse effects for patients who use their shoulders more often. For example, among 59 employees who used a computer for 3 to 6 h daily, 71.1% of them reported there is musculoskeletal discomfort in their neck, shoulder, and back [7]. It has been shown that shoulder stiffness can cause higher absence rates at work and a significant economic burden to the healthcare system [8]. Also, on average, 50% of patients will suffer consistent shoulder pain or stiffness even seven years after the onset of the disease [9].

The labor force population in China has a relatively high percentage of people aged 40 to 59, with more than 30% of people aged 40–49 and more than 20% of people aged 50–59 [10]. Therefore, frozen shoulder causes significant functional disability and pain in a population group consisting of patients who are often middle-aged and working [11]. Although numerous studies have investigated the epidemiology and treatment of frozen shoulder, however detailed studies on its risk factors are still limited. Therefore, the objective of the current study is to investigate in detail the risk factors associated with FS using a retrospective study design to analyze the relationship between gender and diabetes with frozen shoulder further. Chen et al. conducted a logistic multivariate regression analysis among post-treatment breast cancer survivors and found that physical disabilities were prevalent, with 20.3% of patients experiencing limited shoulder joint mobility \* MERGEFORMAT [12]. The study by Cao et al. identified potential risk factors for FS in middle-aged adults within one year following venous infusion therapy. Additionally, the type and frequency of venous infusions may also play a role in the occurrence of FS [13]. Zhang et al. employed logistic regression for a multivariate analysis of the clinical data from 592 patients who had arthroscopic rotator cuff repair. The multivariate analysis did not indicate that a history of smoking was a risk factor for the development of frozen shoulder [14].

Frozen shoulder, a prevalent musculoskeletal condition, profoundly affects patients' quality of life. Investigating its risk factors through rigorous case–control studies is crucial for developing a nuanced understanding of the condition, which in turn is vital for crafting effective prevention and treatment approaches. Understanding the pathogenesis of frozen shoulder, particularly within specific geographic regions, helps to pinpoint vulnerable populations. Variables such as age, gender, regional variability, seasonal patterns of incidence, and the duration of the disease can provide critical insights into the disease's dynamics, laying the groundwork for informed prevention and management strategies. Early identification and intervention are paramount in reducing the incidence of frozen shoulder. For those at higher risk, proactive measures like timely education and physical therapy can significantly mitigate the likelihood of contraction, alleviating patient distress and enhancing overall well-being. From a public health perspective, decreasing the incidence of frozen shoulder not only boosts patients' quality of life but also eases the load on healthcare systems. Findings from case–control studies offer policymakers a solid scientific foundation for devising more impactful public health strategies and ensuring a more efficient distribution of healthcare resources. In essence, exploring the risk factors associated with frozen shoulder and conducting in-depth case–control analyses are instrumental in enhancing our knowledge of the condition, formulating robust preventive strategies, refining treatment protocols, guiding the development of public health policies, and propelling forward research in this field.

To confirm the possible risk factors related to the development of FS, we have examined 1205 case data obtained in the Xinjiang region of China from the year 2018 to 2023 with the consideration of various factors such as gender, occupation, place of residence, marital status, age, seasons of disease onset, duration of illness, etiology, medical history, sleep quality, smoking and alcohol consumption, constipation, family history, muscle strength, etc. This comprehensive analysis will help us to determine those factors related to the development of FS, and enable us to improve our understanding and diagnosis of FS, and provide us with more effective prevention and treatment methods for patients.

## Method

### Inclusion criteria

1. Pain in the affected shoulder joint, with significant limitations in movement (flexion < 90°) and consistent degrees of active and passive movement limitations;
2. No significant improvement in shoulder joint mobility after conservative treatment.
3. Patients with frozen shoulder supported by imaging and laboratory data such as X-ray, CT, and MRI.

### Exclusion criteria

1. Exclusion of patients with systemic lupus erythematosus or other specific medical conditions that could potentially interfere with the assessment or management of shoulder pain;
2. Exclusion of patients with incomplete medical records, precluding a precise case definition;

### General information

According to the above inclusion and exclusion criteria, 1205 cases of patients who visited the hospital from January 2018 to June 2023 were collected and included in this study. Among them, 573 patients diagnosed with frozen shoulder were selected as the observation group, and 632 age-gender matched patients were randomly chosen as the blank control group. In the control group, there were 163 males and 469 females, while in the observation group, there were 189 males and 384 females. The average age of the control group was 58 years old, and the average age of the observation group was 59 years old.

### Evaluation indicators

Gender, occupation, place of origin, marital status, age, season of onset, duration of illness, cause of disease, surgical history, hypertension, diabetes, respiratory system diseases, knee joint disease, hyperlipidemia, cardiovascular and cerebrovascular diseases, cervical spondylosis, lumbar disc herniation, rheumatoid arthritis, hyperuricemia, sleep status, smoking and drinking alcohol, and whether constipation occurs.

### Statistical methods

SPSS 26.0 software was used in all statistical analyses. For measurement data that adhered to the normal distribution, the mean ± standard deviation $\overline x\pm s$ was used to represent the data, and the t-test was employed for comparison. For data that did not follow the normal distribution, the median and interquartile range were used to represent the data, and the rank sum test was applied. Count data were expressed as composition ratio or rate (%) and compared using the χ^2^ test. Graded data were also expressed as composition ratio or rate (%) and compared using the rank sum test. Influencing factors were analyzed using a multifactor Logistic regression model, and a *P*-value of less than 0.05 was considered statistically significant.

## Results

### Single-factor logistic regression analysis

As shown in Table 1, single-factor logistic regression analysis revealed that the observation group has significantly more females, more patients with diabetes, knee joint disease, constipation, and poor sleep quality than the control group (*P* < 0.05). It also shows that there were no significant differences between the two groups in terms of occupation, place of origin, marital status, age, duration of illness, season of disease onset, etiology, surgical history, hypertension, respiratory diseases, hyperlipidemia, cardiovascular diseases, cervical spondylosis, lumbar disc herniation, rheumatoid arthritis, hyperuricemia, smoking, and alcohol consumption history (*P* > 0.05).

**Table 1:** Results of single-factor logistic regression analysis

Indicator		Control group	Observation group	χ^2^/t value	*P* value
Gender	Male	283	173	27.184	0.000
	Female	349	400		
Nature of work	Brain workers	399	374	1.188	0.552
	Manual labor	194	160		
	Other	39	39		
Place of origin	Southern Xinjiang	303	285	3.068	0.216
	Northern Xinjiang	304	255		
	Eastern Xinjiang	25	33		
Marital status	Married	521	467	2.925	0.403
	Unmarried	11	7		
	Widowed	100	99		
Age		58(11)	59(10)	-1.848	0.065
Disease duration (Months)		24 (12, 76.5)	24 (6, 72)	1.891	0.059
Season of onset	Spring	165	141	2.823	0.420
	Summer	132	133		
	Autumn	94	70		
	Winter	241	229		
Causes	Exhaustion, cold	499	463	5.499	0.064
	Old disease	104	72		
	Falls (fracture dislocation)	29	38		
History of surgery	No	247	218	0.136	0.712
	Yes	385	355		
Hypertension	No	386	359	0.317	0.574
	Yes	246	214		
Diabetes	No	514	357	54.296	0.000
	Yes	118	216		
Chronic bronchitis, asthma	No	524	497	3.399	0.065
	Yes	108	76		
Knee disease	No	445	427	2.537	0.111
	Yes	187	146		
Hyperlipidemia	No	615	566	3.319	0.068
	Yes	17	7		
Cardiovascular disease	No	429	417	3.443	0.064
	Yes	203	156		
Cervical spine disease	No	448	377	3.609	0.057
	Yes	184	196		
Cerebrovascular disease	No	599	552	1.701	0.192
	Yes	33	21		
Lumbar disc herniation	No	466	422	0.001	0.973
	Yes	166	151		
Rheumatoid arthropathy	No	514	483	1.849	0.174
	Yes	118	90		
Hyperuricemia	No	628	570	0.062	0.803
	Yes	4	3		
Sleep	No	232	274	29.190	0.000
	Yes	400	299		
Smoking	No	578	537	2.224	0.136
	Yes	54	36		
Drinking alcohol	No	575	537	3.159	0.075
	Yes	57	36		
Constipation	No	530	409	27.220	0.000
	Yes	102	164		

### Multifactor logistic regression analysis results

In the univariate analysis, several factors were found to be statistically different from the healthy group at a significance level of *P* < 0.05. These factors included female gender, diabetes, knee joint disease, constipation, and poor sleep quality. In the final multivariate model, four variables were retained: gender, history of diabetes, sleep quality, and constipation. Among these, diabetes (odds ratio [OR] = 2.349, 95% confidence interval [CI]: 1.790–3.082), gender (OR = 1.765, 95% CI: 1.375–2.265), poor sleep quality (OR = 1.716, 95% CI: 1.349–2.183), and constipation (OR = 2.150, 95% CI: 1.608–2.876) were identified as risk factors for the development of frozen shoulder(Table 2).

**Table 2:** Results of multifactor logistic regression analysis

	β	S_b_	Wald*X*^2^	*P*值	OR	95% CI	
Sex	0.568	0.127	19.896	0.000	1.765	1.375	2.265
History: Diabetes	0.854	0.139	37.905	0.000	2.349	1.790	3.082
Sleep	0.540	0.123	19.381	0.000	1.716	1.349	2.183
Constipation	0.766	0.148	26.617	0.000	2.150	1.608	2.876

**Fig. 1:**
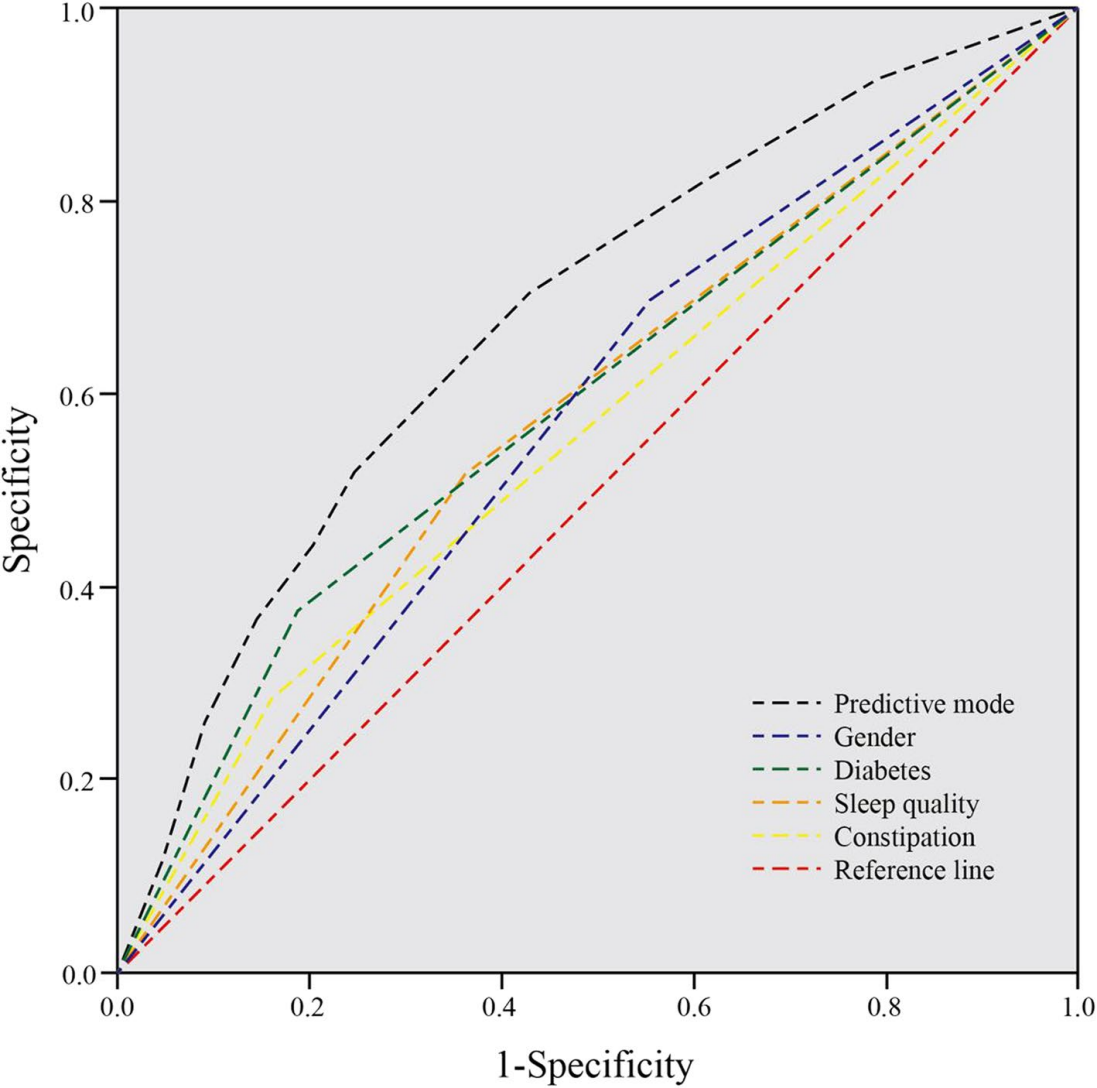
ROC curve of the predictive model and the various factors

Multivariate logistic regression analysis revealed that diabetes and gender are significant factors affecting the risk of FS. The ROC curves of the predictive model and each factor was compared as shown in Fig. 1. The area under the ROC curve (AUC) of the predictive model was 0.682, and the 95% confidence interval (CI) was 0.652–0.712. After a thorough multivariate logistic regression analysis, we identified diabetes and gender as two key factors influencing the risk of frozen shoulder. Specifically, the risk for diabetic patients was significantly higher than that for non-diabetic patients, and in terms of gender differences, female patients were more likely to develop frozen shoulder than male patients. This finding has important implications for clinical practice, potentially aiding doctors in diagnosing and predicting the occurrence of frozen shoulder more effectively.

Furthermore, we developed a predictive model to estimate the likelihood of an individual developing frozen shoulder. The model's AUC of 0.682 suggests that it has moderate predictive power. However, the 95% CI of 0.652–0.712 indicates a degree of uncertainty in the estimate of predictive performance. Consequently, future studies will explore additional potential predictive factors to refine the model and enhance its predictive accuracy. Currently, the model's capability in predicting frozen shoulder is acceptable but has room for improvement. The AUC value of 0.682 indicates a moderate accuracy in distinguishing between patients with and without frozen shoulder, yet it is not ideal. The clinical value of the model will depend on its performance and its ability to assist doctors in making more precise diagnostic and treatment decisions. Overall, this study offers a new perspective for the early identification and treatment of frozen shoulder and has the potential to facilitate the development of more effective clinical management strategies.

## Discussion

### Frozen shoulder

Frozen shoulder, also known as adhesive capsulitis of the shoulder joint, is a common shoulder problem [15]. It is characterized by limited shoulder joint mobility, pain, and stiffness [16]. Frozen shoulder primarily involves inflammation and fibrosis of the soft tissues around the shoulder joint, leading to a restricted range of motion (ROM) and pain [17]. The pathogenesis of frozen shoulder is divided into three stages: the freezing phase, the stiff phase, and the recovery phase [18]. The disease process generally consists of three stages: an initial painful freezing phase, which lasts about 2 to 9 months, during which the patient experiences severe and generalized shoulder pain, particularly exacerbated at night; followed by the freezing phase, which lasts 4 to 12 months, where the pain begins to subside, but the ROM of the shoulder joint is gradually decreased; and finally, the thawing phase, where the ROM of the shoulder joint gradually increases, a process that may take from 5 months to 2 years [19]. The exact cause of frozen shoulder is not yet fully understood, but several risk factors are assumed to be associated with its occurrence. In addition to the factors mentioned in the discussion section regarding gender and diabetes, age, shoulder injuries, prolonged poor posture, and certain systemic diseases can all increase the risk of frozen shoulder.

### Diagnosis of periarthritis of the shoulder

Through literature review [14] we found that the diagnostic terms for cases of unexplained shoulder joint stiffness are as follows: frozen shoulder (31%), stiff shoulder (22%), periarthritis (16%), the Japanese term “Gojukata,” which refers to shoulder joint issues in people aged 50 and older (16%), idiopathic frozen shoulder (6%), primary frozen shoulder (4%), adhesive periarthritis (3%), and others (2%). Conduct radiological studies to rule out other secondary causes of shoulder pain [20]. The diagnosis of frozen shoulder was established through an evaluation of the patient’s medical history and a physical assessment [21].

This may be due to the differences in the understanding and classification of shoulder diseases between Western and Chinese medicine. In Western countries, frozen shoulder and adhesive capsulitis are more common shoulder disease diagnoses [22]. In China, periarthritis is a broader concept that includes frozen shoulder, adhesive capsulitis, and other shoulder diseases. This may be because Chinese medical research and clinical practice emphasize a holistic view of the disease and dialectical treatment, thus categorizing some similar shoulder diseases using the common term periarthritis during diagnosis and treatment. The diagnosis of frozen shoulder in this study is based primarily on clinical findings, including limitation of ROM and pain in the shoulder. Key diagnostic steps include a detailed patient history, particularly the onset time of pain and decreased ROM, and a thorough physical examination to look for tenderness and muscle wasting in the shoulder area. Imaging has played a supporting role in this diagnosis process to rule out other causes and evaluate shoulder structures. These diagnostic processes ensure accurate identification and effective treatment of frozen shoulder [23].

**Fig. 2:**
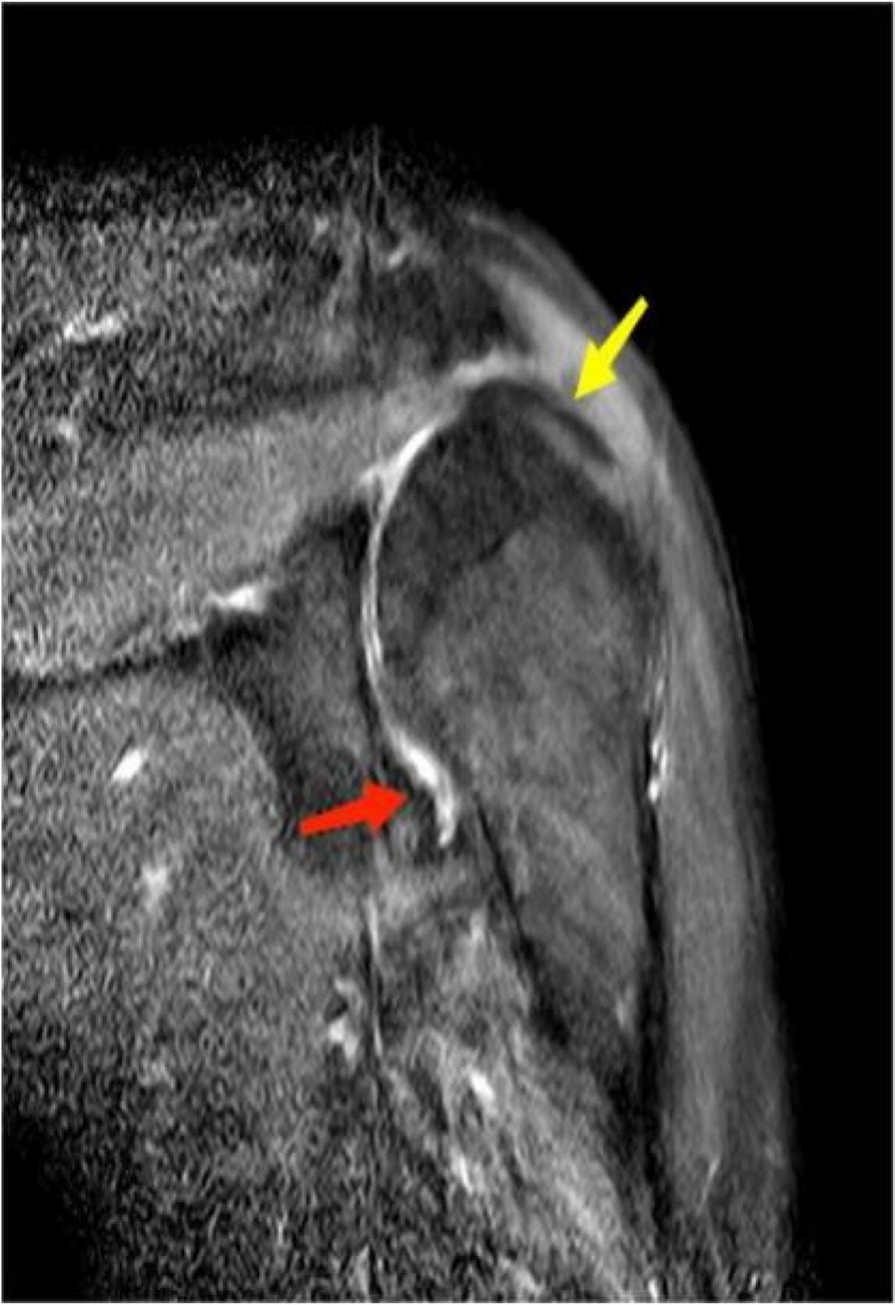
MRI image, Coronal T2WI. Male, 69 years old

**Fig. 3:**
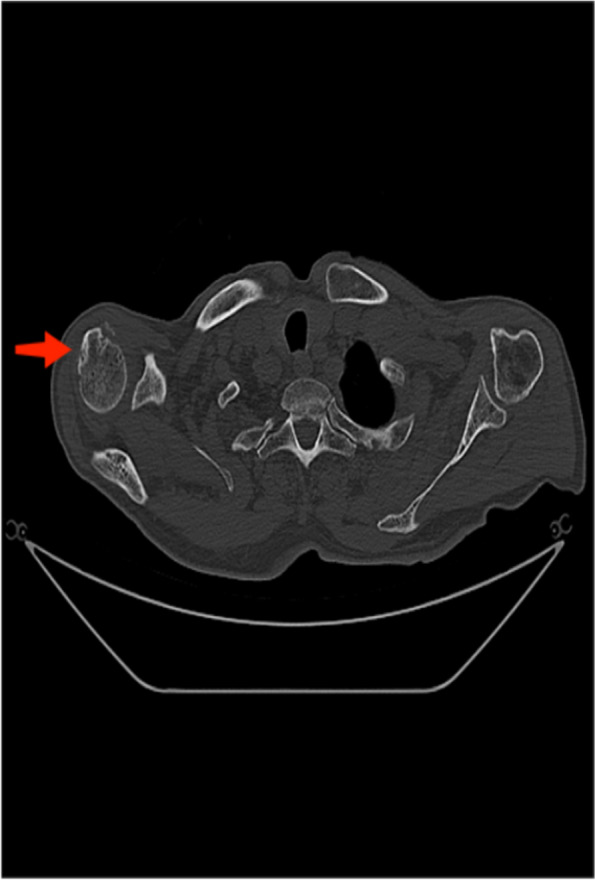
Plain CT scan of the humerus. Male, 80 years old

**Fig. 4:**
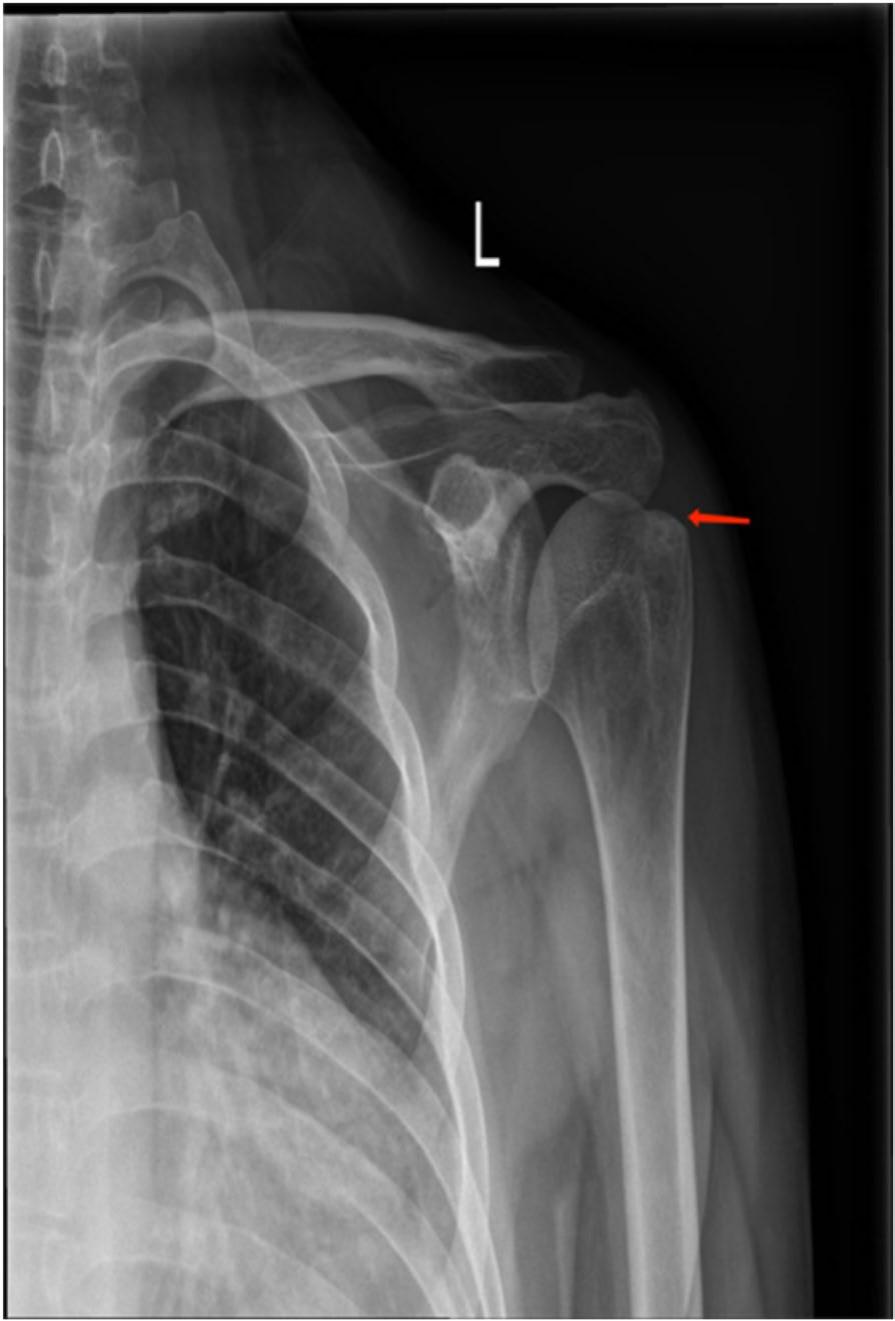
X-ray of the left humerus. Male, 50 years old

MRI, CT, and DR imaging results of patients' shoulder joints are shown in Figs. 2, 3, and 4. In Fig. 2, MRI imaging showed left shoulder joint bone hyperplasia, joint space narrowing, and uneven signal of the humeral head, suggesting the possibility of osteoarthritis. In particular, the mixed high signal on T2WI and the high signal on the fat suppression sequence may be related to degenerative changes and inflammatory responses in the articular cartilage. The increased signal of the supraspinatus tendon, the high signal on T2WI, and the thickening of the shoulder joint capsule further support the hypothesis of muscle and soft tissue injury or inflammation.

CT scan results (Fig. 3) showed right humeral head bone hyperplasia and sclerosis, with rough edges accompanied by osteophyte formation, consistent with osteoarthritis's typical characteristics. The cystic changes below the joint surface may be due to the destruction of the articular cartilage and local osteoporosis. Notably, although there was bone hyperplasia, there was no significant narrowing of the joint space, which may be related to the early stage of the disease.

DR imaging (Fig. 4) further confirmed the bone hyperplasia of the left humeral greater tuberosity, increased bone density, and slight swelling of the surrounding soft tissue, which may be related to local inflammatory reactions or muscle injuries.

Comprehensive imaging results suggest that the shoulder joint lesions in this study may be related to senile osteoarthritis. The progression of the disease can lead to limited joint movement and pain, affecting the patient's quality of life. Therefore, early diagnosis and treatment are crucial for alleviating symptoms and delaying disease progression. Future studies should explore more effective treatment strategies for such lesions and how to monitor disease changes through non-invasive methods.

Due to the lack of large-scale data, the study could not thoroughly compare and analyze the similarities and differences in the diagnostic criteria and methods for frozen shoulder between Western countries and the Xinjiang region of China. This involves differences in diagnostic techniques and includes variations in disease awareness, treatment strategies, and patient education. These factors may affect the study results and limit our understanding of the pathological mechanisms and treatment methods for frozen shoulder. Therefore, future studies need to be conducted on a larger scale and in more regions in order to gain a more comprehensive understanding of the current state of the global diagnosis and treatment of frozen shoulder and to inform the development of more generalized diagnostic and therapeutic protocols.

Hirata J et al. found that female patients with frozen shoulder experience higher pain intensity and lower self-efficacy [24], similar to our findings in this study. Jacob L et al. studied [25] the prevalence and risk factors of frozen shoulders in older adults in Germany using a cross-sectional survey with questionnaires and shoulder examinations and obtained a similar prevalence to that we found in the Xinjiang population of China in this study. The results showed that the prevalence of shoulder joint adhesion was 4.6%, with females and the right shoulder joint being more commonly affected, similar to our results. In addition, factors such as obesity, diabetes, hypertension, and cardiovascular disease were found to be associated with the development of frozen shoulder. These results may provide some guidance for the prevention and treatment of frozen shoulder.

Dietary and regional characteristics may indirectly affect the risk of frozen shoulder by influencing the health status of individuals [26]. In the Xinjiang region, due to the cold climate and occupational characteristics, individuals may be more prone to muscle tension and poor blood circulation, which will increase the prevalence of frozen shoulder. Furthermore, the residents of Xinjiang mainly consume meat, naan (a type of bread), and dairy products in their daily diet, which is significantly higher in fat and carbohydrates compared to the inland areas of China. The special diet and lifestyle of the Xinjiang region have resulted in higher incidence rates of hypertension, diabetes, and obesity than those in the inland areas [27]. These metabolic diseases may have some association with the development of a frozen shoulder. However, further studies are needed to verify this association and explore the mechanisms by which dietary and regional characteristics affect frozen shoulder.

### Frozen shoulder and diabetes

Recent studies have shown that diabetes may be an independent risk factor for frozen shoulder[28]. Diabetes is a metabolic disease that has been confirmed to be associated with a variety of systemic diseases and pain symptoms \* MERGEFORMAT [29]. The results of our study indicate a significant relationship between diabetes and frozen shoulder. In our sample, the incidence of frozen shoulder among diabetic patients was significantly higher than in the non-diabetic population. This result supports the findings of Cole et al., where over 25% of diabetic patients were diagnosed with periarthritis.

Additionally, it has been confirmed that the incidence of complications and frozen shoulder ranges from 10 to 35%, and these patients have more severe stiffness, which should be actively managed [30, 31]. There was evidence that poor long-term blood sugar control in diabetes patients was associated with an increased incidence of periarthritis, whereas there was no association between the hemoglobin A1c levels of diabetes patients and the incidence of periarthritis [32]. Multiple mechanisms can explain the relationship between diabetes and frozen shoulder. Based on an existing histological study [33], the pathophysiological process of frozen shoulder consists of chronic inflammation and capsular fibrosis, which leads to contracture. It is speculated that the accumulation of advanced glycation end products (AGEs), which cause collagen cross-linking, can explain the fibrosis in the capsule of patients with periarthritis [34]. Diabetes can cause damage to blood vessels and nerves, which may lead to abnormalities in blood supply and nerve conduction around the shoulder joint [35]. Tendon dysfunction may be due to chronic inflammation caused by diabetes. In diabetes patients, the long-term release of inflammatory mediators such as tumor necrosis factor α (TNF-α) and interleukin-6 (IL-6) triggers a series of inflammatory reactions, leading to the accumulation of extracellular matrix components such as collagen and ultimately to tendon fibrosis and dysfunction, which can lead to the development of frozen shoulder [36–39]. Diabetic patients often have chronic hyperglycemia and diabetes-related complications, such as diabetic neuropathy and diabetic myopathy. The development of diabetic myopathy is independent of other diabetes complications [40]. These complications can cause functional abnormalities in the muscles and nerves around the shoulder joint, increasing the risk of frozen shoulder [41].

Additionally, diabetic patients often have a systemic chronic inflammatory state [42], which may promote the occurrence of frozen shoulder by affecting the inflammatory response and immune function of the shoulder joint. Abnormal secretion of inflammatory factors and cytokines may play an essential role in the association between diabetes and frozen shoulder \* MERGEFORMAT [43]. There is some correlation between frozen shoulder and diabetes. Diabetic patients are more easily to develop frozen shoulder, which may be one of the complications of diabetes, and consequently frozen shoulder can affect the quality of life and treatment outcomes of diabetes patients. At present, we cannot define the exact correlation between patient blood sugar thresholds and the development of frozen shoulder. Therefore, it is important for diabetes patients to prevent and treat frozen shoulder on time. At the same time, diabetes patients should also strengthen blood sugar control to reduce the risk of frozen shoulder.

It should be noted that the results of our study indicate that diabetes is an independent risk factor for frozen shoulder, but this does not mean that all diabetes patients will develop frozen shoulder. Other genetic, environmental, and behavioral factors may also play a role in the occurrence of frozen shoulder. Therefore, diabetes patients should pay attention to controlling blood sugar levels and actively improving their lifestyle to reduce the risk of frozen shoulder.

### The relationship between frozen shoulder and gender

Gender plays an independent risk factor role in the development of frozen shoulder. Especially females are more prone to develop frozen shoulder than males, and female patients are at a higher risk of developing the condition [32]. It was found that glutamate injected into the masseter muscle produced more intense pain in women than in men [44]. These gender differences may be related to physiological and psychological factors, such as gonadal hormones and central hyperexcitability [45]. Additionally, estrogen may have a pro-inflammatory effect, which may also explain why women are more prone to pain problems such as neck and shoulder pain [46]. These findings help us better understand the role of gender differences in pain regulation and provide guidance for the development of gender-specific pain treatment methods.

Females may have differences in perceiving muscle pain. Studies have shown that women may be more likely to perceive pain than men when exposed to repeated stimuli due to the higher levels of central hyperexcitability in women. It may indicate that the central nervous system of women is more easily stimulated and leads to more sensitive pain perception [47]. In comparison, male gonadal hormones have inhibitory and adaptive effects that can reduce the response to harmful stimuli [48]. Women have a lower pressure pain threshold (PPT), meaning they may be more likely to feel pain than men when exposed to the same pressure stimulus as men [49]. Although women have more trapezius muscle than men, there is no gender difference in pain sensitivity to pressure. However, the posterior lateral neck and masseter muscles have shown gender differences, suggesting that gender differences in pain sensitivity to pressure may be related to muscles in different locations [50, 51]. It has been shown that women are more likely to be affected by temporal lobe development, which may be one of the factors contributing to the common occurrence of muscle pain in women [52]. The incidence of neck and shoulder pain is also higher in women [53]. It has been reported that women may bear more shoulder burdens and stress in work and daily life, such as long periods of computer use, lifting heavy objects, and carrying infants. These activities can lead to muscle tension, poor posture, and overuse of the shoulders and increase the risk of periarthritis [54]. The Chinese government strives for employment equality in the labor market, yet women tend to assume a greater share of family responsibilities, including childrearing, eldercare, and domestic tasks. The dual transition of enduring long work hours followed by an equivalent amount of time spent on household chores can lead to a higher incidence of work-family conflict for women, thereby exacerbating the adverse effects of prolonged working hours on them [55]. The social roles of women may also have an impact on the risk of periarthritis in women. Women have more responsibilities and pressures at work and in the family, and prolonged periods of intense work may lead to physical fatigue and poor posture, increasing the incidence of periarthritis [56]. Our study has shown that women are more likely to develop frozen shoulder compared to men and that female patients are at a higher risk of developing the condition. This result is consistent with the conclusions drawn from previous studies [57], and emphasizes the importance of gender in the development of frozen shoulder. There are several possible mechanisms for why gender is related to the frozen shoulder, as discussed above.

### Frozen shoulder and emotional connection

In addition to the limitation of shoulder function, the pain of frozen shoulder may also negatively affect the emotional state of patients. The main characteristics of frozen shoulder are a restricted range of motion in the shoulder joint and chronic pain around the deltoid insertion, which severely affects the patient's limb function, work capacity, and quality of life [58]. Pain is closely related to emotions and can cause physical discomfort and suffering, which in turn affects an individual's psychological state [59]. The pain of frozen shoulder may lead to anxiety and depression in patients. Long-term pain and limited shoulder joint function bring inconvenience to the patient's daily life, making them feel powerless and frustrated. This persistent negative emotion may further exacerbate the perception of pain, creating a vicious cycle [60]. The pain of frozen shoulder is not only a problem in itself but may also lead to other diseases, such as negative emotional states, depression, anxiety, insomnia, and poor sleep quality. These may interfere with patients' motivation to participate in rehabilitation and their overall health [61]. The shoulder pain and decreased mobility caused by frozen shoulder may reduce overall physical activity in daily life [62]. Side effects of some medications for frozen shoulder may also be related to constipation. For example, nonsteroidal anti-inflammatory drugs (NSAIDs) are a commonly used medication for frozen shoulder treatment, but they are thought to cause problems such as gastrointestinal distress and constipation [46]. Pain from a frozen shoulder can also hurt social and interpersonal relationships. Patients may find it difficult to perform normal physical activities such as shaking hands, hugging, or lifting heavy objects due to pain, which can cause them to avoid social activities, develop feelings of loneliness and inferiority, and even isolate themselves from friends and family, further exacerbating emotional problems. Also, the patients can not sleep well due to pain [63]. Because the pain can cause emotional problems, emotional issues may also increase the perception of pain, and thus, when treating frozen shoulder, attention should also be given to the patient's mental health in addition to the pain itself [64]. In addition to physical therapy, psychological counseling, and support are also crucial treatment modalities. By offering emotional support and coping strategies, patients can adjust their mental attitude, alleviate anxiety and depression, and enhance their ability to manage pain. The onset of frozen shoulder results in shoulder pain and limited mobility and may also negatively impact a patient's emotional and physical health as follows: Firstly, due to the presence of pain and movement restrictions, patients may experience anxiety, frustration, and anger, which can affect their emotional state. Secondly, frozen shoulder is often accompanied by issues such as constipation and insufficient sleep, which can further exacerbate emotional distress. Through the analysis of cases over the past five years, we have found that gender and diabetes play significant roles in the incidence of frozen shoulder. Clinically, this finding reminds physicians to consider gender and diabetes factors when evaluating and treating frozen shoulder. Closer attention and preventive measures should be given to female patients and those with diabetes to reduce the risk of developing frozen shoulder. However, it should be noted that this study has some limitations. The study utilized a retrospective design, which may be subject to information bias and omission. The findings can be applied only to a specific patient cohort, and further studies are needed to verify whether they can be generalized to other populations. In future studies, we suggest a larger-scale, multicenter prospective study to validate the effect of gender on frozen shoulder further and to explore the mechanisms in physiology and behavior deeply. This will help deepen the understanding of the pathogenesis of frozen shoulder and provide more targeted strategies for individualized prevention and treatment. The results of this study indicate that gender and diabetes are independent risk factors for frozen shoulder, with women being more susceptible to the condition than men and individuals with diabetes being at a higher risk. These findings have significant implications for clinical practice and highlight the importance of considering gender factors in assessing and treating frozen shoulder. Future studies should further explore the physiological and behavioral mechanisms, including gender differences, to provide more effective preventive and treatment strategies.

Understanding the role of gender in the development of frozen shoulder could lead to more personalized approaches to prevention and treatment. For example, healthcare providers might recommend specific exercises or interventions tailored to the needs of women or individuals with diabetes to reduce the risk of developing frozen shoulder. Additionally, early identification of risk factors could facilitate timely intervention and potentially mitigate the severity of the condition.

As for the behavioral aspects, studies might focus on lifestyle factors that can be modified to reduce the risk of frozen shoulder. The diagnosis and treatment process can include ergonomic assessments to prevent repetitive strain injuries, training with proper posture and movement patterns, and stress management techniques to address the potential impact of psychological stress on shoulder health.

In conclusion, the recognition of gender and diabetes as risk factors for frozen shoulder emphasizes the need for a comprehensive, personalized approach to management that addresses the physical and psychological aspects of FS. Future studies should deepen our understanding of these risk factors and develop evidence-based strategies to prevent and treat FS in various patient populations effectively.

The limitation of this study is that researchers need to explore the potential relationship between blood glucose levels and frozen shoulder further in order to provide more valuable information for clinical treatment.

## Data Availability

The data used to support the findings of this study are included within the article.

## Abbreviations

FS: Frozen shoulder
NSAID: Non-steroidal anti-inflammatory drugs
PPT: Pressure pain
ROM: Restricted range of motion
